# Adaptive Deployable Structure Enabled by Actively Controlled Tensegrity for Space Debris Removal

**DOI:** 10.1002/advs.202408617

**Published:** 2025-02-14

**Authors:** Endong Shang, Ao Li, Md Shariful Islam, Li‐Yuan Zhang, Changyong (Chase) Cao

**Affiliations:** ^1^ School of Mechanical Engineering University of Science and Technology Beijing Beijing 100083 China; ^2^ Laboratory for Soft Machines and Electronics Department of Mechanical and Aerospace Engineering Case Western Reserve University Cleveland OH 44106 USA; ^3^ Advanced Platform Technology (APT) Center Louis Stokes Cleveland VA Medical Center Cleveland OH 44106 USA

**Keywords:** active control, adaptive configuration, deployable structure, space debris, tensegrity

## Abstract

The Earth's orbital environment is increasingly congested with space debris, posing a substantial risk to space operations and safety. Current mitigation strategies are primarily tailored to either small debris, through protective devices, or large debris, via spacecraft deorbiting methods, leaving medium‐sized debris (0.4–10 cm) as a significant unaddressed threat. This study introduces an innovative adaptive deployable structure, utilizing actively controlled tensegrity, designed specifically for the removal of medium debris. The basic configuration and deployment process of the structure is detailed, followed by an analysis of key structural parameters affecting its folding and deployment performance. Additionally, the load‐bearing capacity and impact resistance of the structure when integrated with a mesh fabric are evaluated. The optimal parameters and morphology for effective debris removal are identified, culminating in the construction of a 1:20 scale prototype for experimental validation. This structure not only adapts its configuration based on operational needs but also withstands impacts from space debris, thereby playing a crucial role in enhancing orbital safety.

## Graphical abstract

An innovative adaptive deployable structure leveraging tensegrity principles to capture and remove medium‐ and small‐sized space debris. The structure's active rope control ensures stability across various positions, enabling adaptive deformation. This design not only addresses space debris removal but also supports advancements in deployable antennas and solar panel arrays in space.

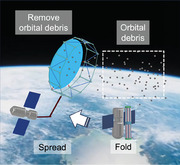

## 1 Introduction

Space debris in Earth's orbit presents a significant threat to space operations and exploration.^[^ ^1^, ^2^, ^3^ ^]^ According to the European Space Agency, by the end of 2020 (Table S1, Supporting Information), there were over 34 000 debris pieces larger than 10 cm, ≈900 000 between 1 and 10 cm, and roughly 130 million between 0.1 and 1 cm in orbit.^[^ ^4^ ^]^ Debris poses different levels of risk depending on its size. For instance, millimeter‐sized particles can damage satellite subsystems, debris over 1 cm may cause satellite malfunction or disintegration, and pieces larger than 10 cm could destroy spacecraft, leading to further debris generation.^[^ ^5^ ^]^ The highest risk of collision occurs in low Earth orbit due to its dense satellite population and higher relative speeds compared to geosynchronous and medium Earth orbits.^[^ ^6^, ^7^ ^]^

While small debris under 0.4 cm poses the highest risk due to its abundance, most are adequately managed by satellite protective measures.^[^ ^8^, ^9^, ^10^ ^]^ Conversely, large debris pieces over 10 cm are fewer and can be actively tracked and avoided.^[^ ^11^ ^]^ However, medium‐sized debris, ranging from 0.4 to 10 cm, represents a critical challenge; they are numerous enough to pose a significant risk and large enough to elude effective passive defense.^[^ ^12^ ^]^

Current debris removal technologies mainly focus on large objects and involve strategies like robotic manipulators for satellite dismantling or electrodynamics tethers for altering orbital paths.^[^ ^13^, ^14^, ^15^ ^]^ Though these technologies are advanced, they suffer from high costs, limited reusability, and operational challenges like tether manipulation. Efforts to address the medium debris problem include proposals for spraying liquids or particles to alter debris orbits and concepts for deployable structures that could capture debris in bulk.^[^ ^12^, ^16^ ^]^ However, these solutions face issues such as poor control over dispersed particles, potential risks to operational satellites, and challenges with deploying structures that cannot be retracted once in orbit. Given these complexities, the need for innovative solutions to remove medium‐sized space debris remains a pressing concern, underscoring the urgency of developing effective and sustainable debris mitigation strategies.

Deployable and foldable structures, extensively applied in aerospace since the 1960s, are recognized for their high strength‐to‐weight and stiffness‐to‐weight ratios, minimal thermal expansion, and excellent geometric stability. These features make them particularly suitable for developing new solutions to the space debris problem, allowing for compact storage and transport while supporting substantial loads when deployed.^[^ ^17^, ^18^, ^19^, ^20^ ^]^ Such structures represent a promising direction for enhancing space debris management technologies.

**Figure 1:**
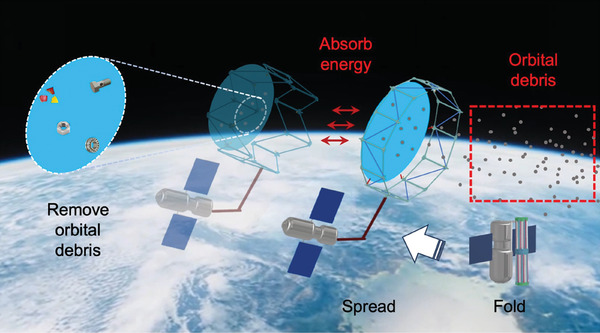
Schematic illustration of the adaptive deployable structure used for space debris removal in the lower orbits. The proposed structure is delivered into space in a folded state with minimal volume for easy transport. In the target orbits, it will be unfolded to form a large dome for small debris collection. It will be able to actively control its size and shape to avoid the possible collision with operating space aircrafts, satellites, and objects to avoid possible damage.

Tensegrity has the characteristics of prestress, self‐adaptation, and self‐balance,^[^ ^21^, ^22^ ^]^ which is often used in the design of folding structures. In this study, we introduce an advanced adaptive deployable tensegrity structure designed to address the challenge of space debris in Earth's orbit, specifically targeting small and medium‐sized fragments (**Figure** **1**). At the heart of this system is a large, ring‐shaped truss coupled with a mesh fabric, engineered to capture space debris with relatively low kinetic energy. For small‐sized debris, the structure's fabric directly collects the fragments. For medium‐sized debris with higher kinetic energy, the structure is designed to passively contract, activating an energy absorption mechanism. This process effectively decelerates the debris, altering its trajectory and leading to its incineration upon re‐entry into Earth's atmosphere, thus neutralizing its threat. However, space also contains large‐sized debris with high kinetic energy levels that could compromise the structural integrity of the deployable system. To protect against such threats, the structure is equipped with the capability to actively retract, allowing it to dodge potential collisions. This retraction feature is enhanced by strategic orbital adjustments designed to avoid large objects, ensuring the structure's durability and operational longevity.

## 2 Results

### 2.1 Design and Analysis of Adaptive Deployable Structure

**Figure 2:**
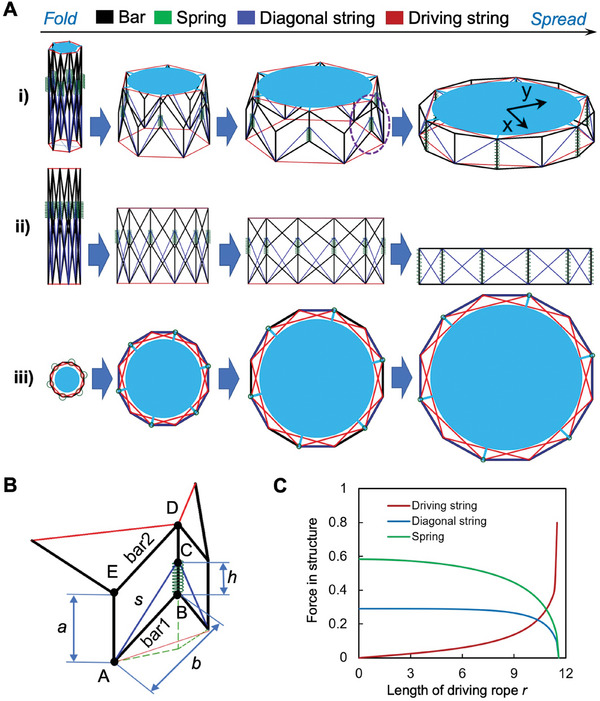
Folding and unfolding process of the proposed adaptive tensegrity structure. A) Deployment Dynamics. (i) The deployment begins as the motor tightens the red driving string, causing the structure to contract progressively. Upon the release of the driving rope by the motor, the green springs exert force, propelling the structure to unfold. (ii) Deployment Diagram (Front View) illustrates the sequential stages of the structure's deployment from a frontal perspective, highlighting the strategic contraction and expansion phases that define its operational cycle. (iii) The Deployment Diagram (Top View) complements the frontal view by providing a holistic understanding of the structure's spatial transformation during deployment. B) Structural and Mechanical Dynamics. (i) Basic Folding Unit. These units are the fundamental building blocks that enable the modular and scalable nature of the structure's design. (ii) Flexible part Force Dynamics: the variations in force experienced by the driving string, spring, and diagonal string during the operational cycle. This insight is crucial for understanding the mechanical stresses at play and ensuring the structure's reliability and longevity under different deployment scenarios.

Given the structure's configuration, with *v* = 6 repeating deployable units, **Figure** **2**A graphically depicts the structural unfolding process, showcasing the synergy between the structure's critical components. Figure 2B shows one of these pivotal units. To systematically examine the unfolding process, we utilize a Cartesian coordinate system. The origin is strategically placed at the center of the circumscribed circle that encompasses the structure at its base. This geometrically informed choice enables a precise analysis of the structure's dynamic behavior during deployment.

#### 2.1.1 Generalized Coordinates

##### Driving String Length (*r*)

The length of the driving string is a crucial parameter that influences the degree of contraction or expansion of the deployable unit. By modulating *r*, we exert control over the spatial configuration of the structure, guiding its transition from a folded to an unfolded state.

##### Spring Length (*h*)

The spring's length acts as a counterbalance to the driving string, providing the necessary elastic force for unfolding. The interplay between *r* and *h* dictates the kinetic energy stored and released during the deployment, shaping the trajectory of each deployable unit.

In the supporting information, we describe mathematically the position relationships of the points in the deployable unit with respect to the selected coordinate system during the expansion process.

The establishment of a theoretical model for the structure is an essential step in quantitatively analyzing its behavior and performance, particularly during the unfolding process. With the original lengths of the compression spring and inclined string designated as *H* and *S*, respectively, and their lengths during unfolding designated as *h* and *s* (*s* = ∣A*_i_* C*_i_* ∣), we can calculate the energy stored in these components. The spring constant and string constant are denoted as *k_h_* and *k_s_*, respectively. Then, the energy stored in both the compression spring and the inclined string can be calculated:

(1) $$E_{h}\left(h\right)=\int_{H-b}^{H-h}k_{h}\;\cdot \left(H-h\right)dh$$

(2) $$E_{s}\left(r,h\right)=\int_{\sqrt{a^{2}+b^{2}}}^{s-S}k_{s}\cdot \left(s-S\right)\;ds$$

If the netting's effects are negligible, focusing solely on the compression spring and inclined string offers a simplified yet insightful perspective into the energy dynamics of the tensegrity ring. The total energy within the structure, in this scenario, is derived from the potential energy stored in these two components:

(3) $$E_{A}\;\left(r,h\right)=6E_{h}\left(h\right)+12E_{s}\left(r,h\right)$$

The partial derivatives of the total energy of the structure with respect to the driving string length *r* and the compression spring length *h* are zero in the stable state:

(4) $$\left\{\begin{array}{c} \frac{\partial E_{A}}{\partial r}=0\\ \frac{\partial E_{A}}{\partial h}=0 \end{array}\right.$$

We can accurately determine the positional states of each point within every unit throughout the unfolding process. By incorporating these calculated positions into Equation (3), it becomes possible to trace the structural energy changes during unfolding. Given that the work in this context is solely performed by the red driving string, analyzing the derivative of the total energy relative to the driving string's length enables us to assess the variations in force that the driving rope experiences.

In the design, we embrace the principles of tensegrity to imbue our structure with unparalleled flexibility, stability, and a robust capacity for load‐bearing. The foundation of this design is a circular truss configuration, wherein traditional tension elements are innovatively replaced by elastic strings. This pivotal modification not only augments the structure's capability for seamless folding and unfolding but also significantly enhances its resistance to impacts. As a direct consequence of these design choices, there is a notable reduction in the overall mass of the structure, which translates into reduced transportation costs for aerospace missions. The structure comprises four key elements:

- The **black frame bars**, which establish the fundamental framework of the structure, guide its expansion along a pre‐defined trajectory, ensuring precise and controlled deployment.
- The **green springs**, in conjunction with the **blue diagonal strings**, act as the driving forces behind the unfolding process. Their elasticity is not just a design feature but a functional necessity, providing the structure with its exceptional impact resistance capabilities.
- The **red driving string** serves as the powerhouse of the folding mechanism. Controlled by a motor, this string allows the structure to stabilize in any required intermediate state, offering adaptability and precision in deployment scenarios.

In terms of the structure's overall performance, our analysis concentrates on three key areas: the deployment ratio, the capability for repeated deployment, and resilience against impact forces. The determinants of these performance metrics include the lengths of the horizontal and vertical bars, the initial length and elastic modulus of the spring, the original length and stiffness of the diagonal string, variations in the force exerted by the driving string (Figure 2C), and the type of mesh fabric utilized. The influence of these variables on the structure's functionality and efficiency will be exhaustively explored and elucidated in the following sections.

### 2.2 Effect of Structural Parameters on its Performance

The main components of our structure are designed to bear axial loads. Through specialized node processing, the cross bars experience compression, while the strings are subjected to tension. The forces and stress in these components are directly proportional to their cross‐sectional area. Therefore, the following analysis focuses primarily on the forces within these components, as this is effectively equivalent to conducting a stress analysis.

The dimensions of the horizontal and vertical bars are pivotal in determining the morphology of our tensegrity ring, influencing both its height in a folded state and its extent in an unfolded state. These dimensions are intrinsically linked to the structure's capacity to resist impacts and absorb energy, making the discussion of bar lengths crucial. Using a regular dodecagon as an example, our design incorporates a tensegrity ring that forms a double‐layered regular dodecagon shape when fully expanded, with vertical bars connecting corresponding nodes between the upper and lower layers. Notably, in this tensioned structure, the bearing capacity of the rods is significantly greater than that of the strings. Therefore, in the following theoretical analysis, we consider the rods and the frame they form to be rigid. The structure comprises 12 vertical bars and 24 horizontal bars, the latter being twice the number of the former. This configuration underpins the structure's resilience and energy absorption efficiency, notably during the unfolding process, where potential energy arises from the spring's compression and the string's extension. The greater this potential energy, the more external energy the structure can absorb, which is beneficial.

**Figure 3:**
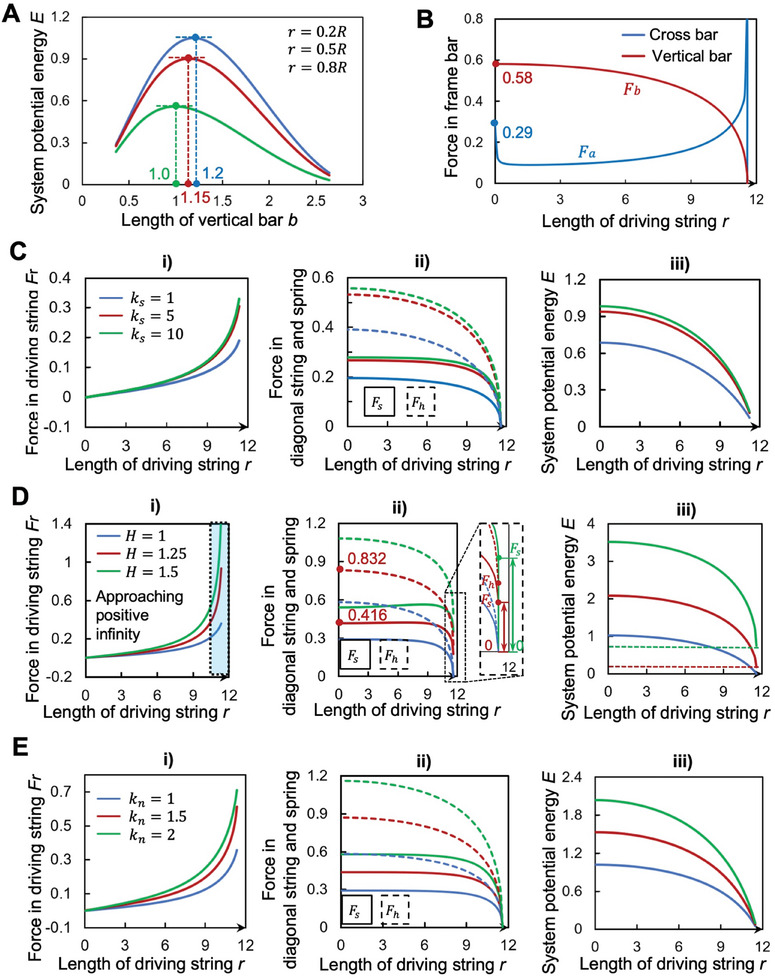
Analysis of structural parameters. A) Influence of the lengths of horizontal and vertical bars on structural performance. Controlling the total mass of the structure (2*a*+*b* = 3), varying the value of vertical bar b. The structure's energy is maximized when b is between 1 and 1.2. B) The change of force in the structural rod. The force in the vertical bar has a strong correlation with that of the compression spring, and its size can be controlled within a certain range. The stress in the crossbar increases sharply when the structure approaches the expansion state, which is a weak part of the structural design. C) Effect of the elasticity coefficient of the diagonal string on the structure. The greater the value of the elastic coefficient is, all the values of the structure, such as the force of the driving strings, the diagonal strings, the springs, and the potential energy of the structure, tend to a stable value, and its value will not increase significantly after exceeding 5. Too small a value will make the structure too loose, and too large a value will make the structure lose some flexibility. D) The effect of the spring length on the structure. (i) the larger the original length of the spring will cause the force in the driving string to increase rapidly, and the excessive force will be unfavorable to the structure when it approaches the complete development; (ii) The value of the original length of the spring will significantly affect the size and change relationship of the diagonal string in the folding process, and too much value will cause its force mutation, which is unfavorable to the structure; (iii) Adjusting the spring length can make the structure have different initial energy and energy absorption effects. E) The impact of compression spring stiffness on the structure. Increasing the stiffness of the compression springs leads to a proportional increase in the overall structural stiffness, which, in turn, enhances various performance parameters of the structure.

To elucidate, we explore the relationship between changes in potential energy and the ratios of bar lengths while maintaining the structure's overall mass constant (*a* + *b* = 3). In **Figure** **3**A, we analyze the variation in potential energy across three intermediate states, under different bar length ratios. It is important to note that the initial lengths of the compression spring and inclined string primarily influence the potential energy's initial value rather than its changing trend. For simplicity, we equate the compression spring's original length with the vertical bar's length, and the inclined string's original length with the distance between points A and D in the fully expanded state. The analysis reveals that potential energy first increases and then decreases as the length of the vertical bar extends, peaking approximately between ratios of 1 and 1.2. A shorter vertical bar results in a greater unfolding ratio and a lower structure height, which is advantageous for the structural design. Consequently, setting the length of the vertical bar to 1, achieving a horizontal‐to‐vertical bar length ratio of ≈1:1, seems most suitable.

For simplicity, let's standardize the elastic constant *k_h_* of the compression spring to a value of 1, and set its original length *H* to *b*. This simplification facilitates a clearer comparison and analysis of effects across different parameters.

In this case (Figure 3B), we initially examined the correlation between stress variations of the cross bars and vertical bars during the folding process of the structure. It was observed that the force in the vertical bar can be controlled within a specific range, whereas the force in the cross‐bar approaches infinity when fully extended. However, there is a similar force variation in the driving rope as in the crossbar, yet our design's rod element has much greater load‐bearing capacity than its rope counterpart. Therefore, for now, we will not delve into discussing the rod and will reserve bearing and stress analysis for later.

In our prior discussions, we highlighted the significance of the original length *S* and the elastic constant *k_s_* of the inclined string as critical parameters for the structural integrity and functionality of our design. Considering the operational dynamics of the inclined string, which is only capable of bearing tensile forces, setting *S* too high would result in a slackened structure upon full deployment, rendering it incapable of sustaining external loads. This state compromises the structure's load‐bearing efficiency. Conversely, an excessively low *S* would place the inclined string and spring in a balanced state of force upon full deployment, making the structure susceptible to passive unfolding even under minor disturbances. Given the potential for high‐frequency impact from spatial debris on the deployable structure's netting, a balance must be struck to avoid premature wear and reduce the structure's lifespan.

As shown in Figure 3Ci,ii, an optimal value for *S* is thus imperative for maintaining structural integrity and functionality. Moving to the discussion of the inclined string's elastic constant *k_s_*, increases in *k_s_* correlate with heightened forces on the driving string, inclined string, and spring, alongside an uptick in the structure's potential energy. As shown in Figure 3Ciii, from an energy absorption viewpoint, a higher *k_s_* is preferable. However, our observations indicate a threshold beyond which additional increases in *k_s_* yield diminishing returns for the structure's performance.

For practical applications, particularly in capturing small‐sized spatial fragments, the structure's design leverages the netting's force in a fully unfolded state to prevent passive unfolding. For medium‐sized fragments, the netting alone may not suffice to dissipate their kinetic energy, risking passive unfolding. This design requires the structure to maintain sufficient prestress, stiffness, and stability when subjected to impact. The prestress in the tensegrity ring primarily originates from the springs and driving strings. This is where a carefully chosen original length and stiffness for the compression spring becomes crucial.

Our analysis, illustrated in Figure 3D, examines the impact of varying the compression spring's original length on the structure's dynamics. A larger original length enhances the structure's potential energy and its resilience against disturbances in a fully unfolded state. Nonetheless, this benefit is capped by practical constraints, as excessively long springs risk structural malfunctions. For example, as shown in Figure 3Di, an excessively long spring would cause the force in the driving rope to tend toward infinity when the structure is fully deployed a clearly undesirable outcome.

Figure 3Dii also reveals that while increasing the spring's original length generally raises the prestress within the structure, a non‐linear response is observed when the length reaches 1.5 times its standard value. This non‐monotonic pattern in the inclined string's force, identified through experimentation, underscores the risk of oversizing the spring's length. Furthermore, the analysis of forces in the driving string suggests a rapid escalation toward infinity as the structure approaches full deployment. To mitigate this, design constraints must be implemented to halt deployment before reaching this critical phase, especially as longer springs necessitate stricter limitations. In summary, while aiming for an optimal spring length that enhances structural stability and resistance to disturbances, excessive elongation proves counterproductive, both from a mechanical stress perspective and in terms of practical deployment restrictions.

Moreover, exploring the stiffness of the tensegrity ring is essential, as it directly depends on the stiffness of the compression springs. As shown in Figure 3E, we analyzed the variation of several parameters under different spring stiffness levels. The data generally indicate a proportional increase in structural stiffness with rising spring stiffness. Therefore, in practical design, it is crucial to select an appropriate spring stiffness based on the load‐bearing capacity of the primary weak components.

### 2.3 Force Analysis and Load Limits of the Tensegrity Ring

In practical operations, the tensegrity ring is engineered to address the challenge of capturing spatial fragments of small and medium sizes. For small‐sized fragments, the structure harnesses the potential energy stored in its springs to either decelerate these fragments or directly collect them. When encountering medium‐sized fragments, should the kinetic energy of these fragments exceed the dissipation capacity of the stored potential energy, the structure is designed to passively contract. This passive contraction allows it to absorb the excess kinetic energy, thereby preventing potential damage.

The design incorporates a strategy for large‐sized fragments as well, which pose a significant threat due to their potential to damage the structure. In such scenarios, an active contraction mechanism is triggered to avoid impact, safeguarding the integrity of the structure. Furthermore, the tensegrity ring is designed for versatility in its deployment. It can be fixed at any intermediate state of expansion, allowing for active control over the degree of expansion and consequently the size of the net. This adaptability is crucial for minimizing damage from sudden impacts by large quantities of fragments, ensuring the structure's long‐term viability.

In operational scenarios, the functionality of the tensegrity ring predominantly hinges on the mesh's ability to decelerate or capture space debris, making the analysis of load and forces exerted on the net critical. Given the diverse orbital paths of space debris in low Earth orbit—including debris in the same orbit moving in opposite directions—the mesh faces impacts from multiple directions, necessitating a comprehensive analysis of force dynamics from various angles.

#### 2.3.1 Analysis of Forces on the Mesh

**Figure 4:**
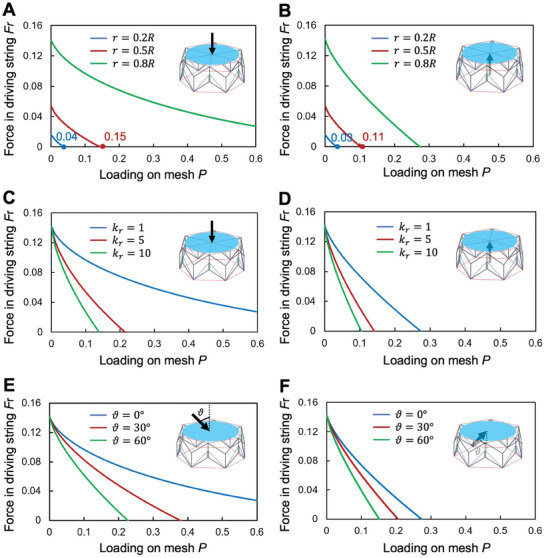
Load on the structure. A) The force change in the driving string after the force is applied to the mesh cloth (the upper end of the force). The bearing capacity of the structure gradually decreases with the contraction of the driving spring. B) The force change in the driving rope after the force is applied to the mesh cloth (the force on the lower end). At this point, the carrying capacity of the structure is slightly weaker than the load applied from the top. C) The influence of different fabric elasticity on the load bearing of the structure (upper load). The greater the elasticity of the fabric, the stronger the load capacity of the structure. D) The effect of different elasticity of the fabric on the load bearing of the structure (the force on the lower end). At this time, the load capacity of the structure is obviously weaker than the upper load. E) The impact of mesh tension angle on structural performance (upper end of force). As the carrying angle increases, the structure's load‐bearing capacity decreases. F) The impact of mesh tension angle on structural performance (lower end of force). In this range, the structure's overall load‐bearing capacity is weaker than at the upper end of the force.

As illustrated in **Figure** **4**A,B, we explored the changes in force within the driving strings when external forces impact the mesh from two distinct directions across various states of expansion. This examination aids in identifying the approximate threshold at which the structure begins to passively contract under external pressure. To simplify the complex dynamics of forces acting on the mesh, we modeled the mesh's forces using five springs emanating from its midpoint, each linking to six ascending nodes. Our findings reveal that the force in the driving strings diminishes regardless of the external force's direction, indicating a passive contraction of the structure beyond a specific critical force level. This threshold increases as the structure expands, yet it also depends on the force's direction, with the critical value for upwardly applied forces generally being slightly higher than that for downward forces.

#### 2.3.2 Material Selection for the Mesh

As illustrated in Figure 4C,D, the choice of material for the netting is pivotal, requiring a balance between elasticity for kinetic energy dissipation and structural integrity. Further investigation is necessary to fully understand how the netting's elasticity influences overall structural performance. We analyzed how the forces within the tether change across three intermediate states under different elastic moduli of the netting. At the same time, changes in the force within the driving strings reflect variations in the structure's stiffness and stability when subjected to external impact. A steeper slope indicates weaker stiffness and stability. Notably, the critical force value for an upwardly applied force remains higher than that for a downward force, with higher elastic moduli correlating to lower critical values. This suggests that a lower elastic modulus may be preferable for the netting, providing sufficient stretch without risking permanent deformation. It will have minimal impact on the structure's stiffness and stability. However, there's a practical limit to the netting's elongation; surpassing this threshold could lead to plastic deformation, undermining the structure's effectiveness.

#### 2.3.3 Analysis of Impact Angle on the Mesh

As shown in Figure 4E,F, in practical applications, the impact load on the mesh can originate from various angles and speeds, rather than being purely vertical or horizontal. This variability makes it essential to examine the effects of different impact angles. We adjusted the load direction to analyze force changes in the driving ropes under both positive and negative loading conditions. Our analysis shows that the threshold for positive loading is higher than for negative loading. Additionally, as the impact angle increases, the threshold decreases. Thus, in practical applications, it is advantageous to align impacts as vertically as possible to maximize load‐bearing capacity. The effect of impact speed will be discussed in the experimental section.

Therefore, while a high elastic modulus might seem advantageous for durability, it is essential to strike a balance. An optimally intermediate value for the netting's elastic modulus should be selected after careful consideration of both kinetic energy dissipation needs and the risk of plastic deformation, ensuring the netting's effectiveness in space debris mitigation without compromising the structure's integrity. To effectively address the challenge of removing small and medium size orbital debris, the material chosen for the net must possess a combination of critical attributes: high‐temperature resistance, significant strength and toughness, adequate elasticity, and minimal weight. In line with our conceptual framework, materials such as Kevlar and Nextel emerge as prime candidates due to their unique properties.^[^ ^23^, ^24^, ^25^ ^]^

### 2.4 Prototype Fabrication and Experimental Characterization

**Figure 5:**
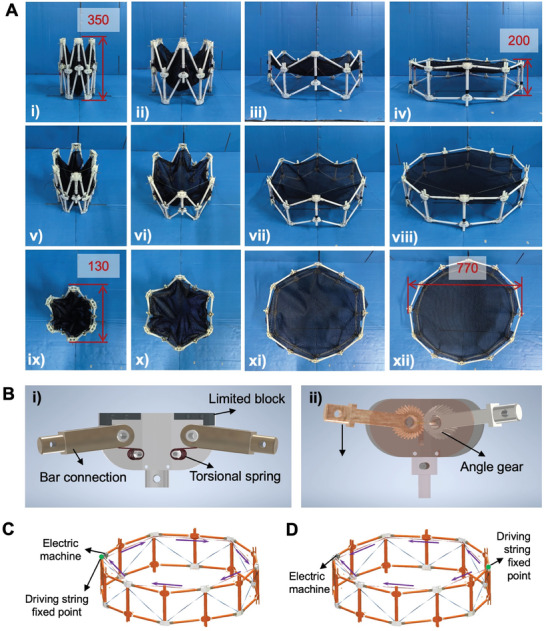
Prototype Design. A) The basic parameters of the prototype and the structure are designed according to 1:20. The structure is ≈350 mm in height and 130 mm in diameter when folded. The height is ≈200 mm, and the diameter is ≈770 mm. The structure can be repeated, and has good robustness and load capacity, basically meet the design requirements. B) Design of key nodes. (i) Five‐bar linkage node. The bar connection is Limited to block the expansion degree of the structure, and the torsional spring makes up for the problem of insufficient development driving force when the structure is folded. (ii) Three‐bar linkage node. The synchronized bar connects the crossbar, and the Angle gear controls the synchronized folding of the structure. C) Unidirectional motor drive system. The driving string is composed of a single rope that wraps around the structure and contracts. D) Bidirectional motor drive system. The driving string consists of two ropes that symmetrically expand or contract on either side of the motor.

To achieve the objectives of on‐demand deployment and the capability for repeated folding, our design integrates a combination of innovative and conventional materials and mechanisms. We utilized 3D‐printed resin for the complex nodes, polyvinyl butyral (PVB) pipes for the structural elements requiring a balance between lightness and stiffness, and tension springs to facilitate the unfolding process. These components are assembled using standard fasteners such as bolts and nuts, ensuring a secure and adjustable structure. As illustrated in **Figure** **5**A, the prototype, scaled down to 1:20 of the actual size, demonstrates both folded and unfolded states. In its compact form, the prototype stands at a height of 350 mm and has a diameter of 130 mm. Upon expansion, it extends to ≈200 mm in height and spans 770 mm in diameter, achieving a notable folding ratio of ≈1:6. Additionally, the design allows for the structure to be arrested at any desired intermediate state of expansion through precise control of the driving string.

To prevent injury during a fatal impact, the tensegrity ring must contract rapidly. Key factors influencing the contraction rate of the tensegrity ring include the truss diameter, number of edges, and the arrangement of the driving strings. For example, as the truss diameter increases, the driving strings become longer, leading to a decrease in the contraction rate. Similarly, as the number of edges increases, the driving cables transition from a polygonal shape to one that closely approximates a circle, increasing their length. However, this length increase is limited and approaches a constant value, equal to the circumference of the circle.

The design and functionality of nodes within the tensegrity ring are critical, acting as the foundation for the structure's extensive capabilities. In our approach, each vertical pole is equipped with two nodes of distinct configurations, designed to meet a variety of operational needs. This ensures the structure's versatility and durability. As illustrated in Figure 5Bi, the five‐bar linkage node, anchored at one end of the vertical supports, plays a starring role in the deployment sequence. Comprising upper and lower cover plates, limiting blocks, bar connectors, and torsion springs, each component contributes to a carefully choreographed operation. The cover plates ensure the structure's integrity, akin to guardians of a castle, while the limiting blocks, acting as directors, define the precise unfolding angles for the horizontal bars, preventing any overextension. The bar connectors, versatile in their function, accommodate horizontal bars of various lengths, adapting seamlessly to the unfolding narrative. Torsion springs, the pivotal elements of this ensemble, provide the necessary momentum to overcome the static equilibrium at the fold, sparking the beginning of the deployment.

Conversely, the three‐bar linkage node, positioned at the opposite end, adopts a more streamlined yet no less essential role. Illustrated on the right in Figure 5Bii, it comprises specialized bar connectors that align the structure's movement through the addition of engaging bevel gears, the number of bevel gears is equal to the number of truss sides, ensuring a symphony of synchronized folding and unfolding across the structure's segments. Despite the uniformity of components among the vertical poles, their differing orientations introduce a compelling twist, ensuring the entire tensegrity structure achieves a balance of forces and an even stress distribution during operation. This nuanced configuration of nodes, with their distinct yet harmonious functions, accentuates the elegance of the design, marrying functionality with the resilience required for space applications.

A significant advantage of this mechanism is its ability to contract with a single motor drive, representing a notable advancement. There are two options for the single‐motor drive. The unidirectional drive design (Figure 5C) uses a single driving string wrapped around the truss in a complete circle, with one end fixed to the motor. The bidirectional drive design (Figure 5D) employs two driving strings that pass through the truss from opposite directions and converge at the motor, with their ends fixed opposite the motor. The bidirectional drive design can double the contraction rate compared to the unidirectional drive design; however, it also requires increased driving power. Therefore, if the motor power is sufficient, the bidirectional drive design is a more efficient choice.

**Figure 6:**
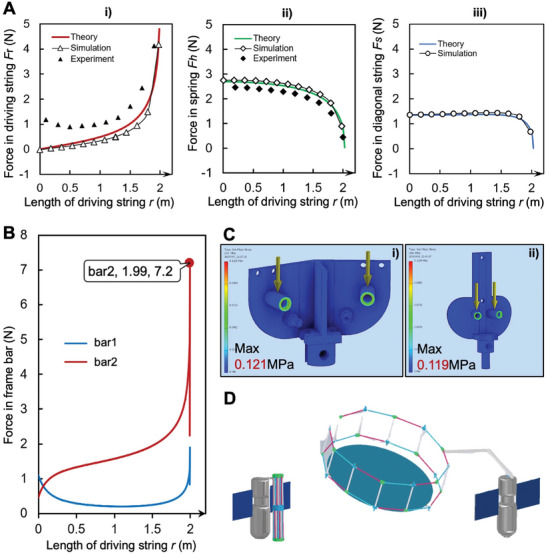
Experimental Results. A) Comparison of simulation and experimental data. (i) Force change of the driving string. The coincidence of theory and simulation results is high, and the experimental data is affected by friction and other factors. (ii) Force change of the spring. The coincidence of theoretical, simulation, and experimental results is high. (iii) Force change of the diagonal string. The results of the experiment and simulation agree well. B) Force changes in the crossbar. The force of bar2 is obviously smaller than that of bar1. When the structure approaches the expansion state, a higher extreme point appears in bar2, and the force in the bar is the largest. C) The stress analysis of bar2's connector shows that the load at the weak point of the connection is ≈0.121 MPa, which is less than the load limit of the material. D) Effect drawing of folding structure space application.

To verify our design's efficacy, we juxtaposed theoretical calculations with simulation outputs and experimental data, as depicted in **Figure** **6**A. This comparison confirms a good alignment between theoretical and simulated results, underscoring the design's soundness. Experimentally, while measuring the force on the driving string is straightforward due to its active control in deployment, gauging the force on the tension springs requires indirect methods such as observing their contraction. Assessing the force on the inclined string poses a greater challenge, leading to experimental data primarily covering the driving string and tension springs. Despite a minor discrepancy in the force observed on the driving string—attributed to the prototype's gravitational effects and friction in the slider mechanism—the experimental findings largely corroborate the theoretical and simulated predictions. This deviation highlights the need for further refinements, particularly in reducing frictional losses and compensating for gravitational influences, to enhance the structure's performance and reliability in future implementations.

Navigating through the complex dance of five‐bar and three‐bar linkage nodes, the structure masters an orchestrated deployment and retraction process. This system meets diverse operational needs and protects against overstretching, guaranteeing smooth transitions past critical points of deployment. Such an approach indeed refines the tensegrity ring's structural and mechanical integrity and markedly boosts its adaptability and durability for real‐world use, signifying a leap forward in the realm of tensegrity‐based engineering.

In the previous analysis, we mentioned that the stress in the crossbar is close to infinity when the structure approaches the fully developed state, so it is necessary to carry out stress analysis on the transverse bar and its connecting parts in this part. Cross bars were identified as potential stress hotspots. This led to a detailed stress analysis of two specific crossbars (bar1 & bar2) within one of the repeating units, particularly under conditions of maximum structural stress (Figure 6B). Conducted via numerical simulations, this analysis revealed that bar2 experience greater stresses than bar1. Nonetheless, the maximum stress recorded during the structure's folding, at 0.12 MPa (Figure 6C), remains safely below the material's yield strength limit, ensuring the structure operates within a safe stress margin. This strategic combination of mechanical durability, functional versatility, and seamless integration with satellite guidance lays a solid foundation for efficacious space debris removal efforts. It represents a proactive and innovative approach to safeguarding the orbital pathway, highlighting our dedication to advancing space exploration and sustainability through ingenious and practical solutions.

The connection points of the mesh with the truss and the driving rope with the truss are identical, with contraction driven by the force directed from these points toward the truss center. As a result, the previously discussed stress analysis also applies to situations where the mesh undergoes passive contraction due to debris impact. However, the impact response is complex and influenced by factors such as material properties and assembly configuration, making stress analysis alone insufficient. Therefore, actual simulations of debris impacts are essential.

In Movie S5 (Supporting Information), we simulated the impact of debris of various sizes on the mesh using weights of 10, 100, 200, 300, and 500 g, with the structural responses documented in Table S2 (Supporting Information). In Movie S6 (Supporting Information), we used a 100 g weight dropped from heights of 0.5, 1.0, 1.5, 2.0, and 2.5 m to simulate impacts at different speeds, with the effects shown in Table S3 (Supporting Information). Applying the principle of momentum conservation, we scaled the momentum of a 500 g weight impacting the mesh and then enlarged the structure by a factor of 20 to match the actual design dimensions. Based on these calculations, we anticipate that this structure can withstand impacts from debris weighing 50 g at a speed of 800 m s^−1^. Additionally, replacing the primary material from resin to aluminum alloy would significantly enhance impact resistance, offering promising potential.

While similar schemes have been proposed, existing research has primarily focused on analyzing the feasibility of these approaches. For example, Takeichi introduced a debris removal strategy using a tethered plate and performed simulations of debris impacts.^[^ ^26^ ^]^ Masaya Nitta conducted simulations to evaluate the effectiveness of a truss for debris clearance.^[^ ^27^ ^]^ Foster extended this concept by combining a circular truss with a mesh and exploring the structure and materials in depth.^[^ ^12^ ^]^ Our current work advances this research by developing a prototype and performing collision simulations, which provides valuable insights for furthering the field.

Our innovative structure, once deployed in space, is adeptly designed for precise unfolding or retraction to address a range of scenarios, including navigating around larger space debris. This dynamic shape adaptability is meticulously controlled through an actuation motor operating the driving strings, emphasizing the necessity for collaboration with a satellite. This collaboration ensures not just a continuous power supply but also provides the ability to fine‐tune the structure's orientation and trajectory as needed. The integration of the structure with a satellite for both power and directional control significantly boosts its adaptability and operational efficiency in the space environment. During its journey to the designated orbit, the structure stays compactly folded, securely held by the tension from the satellite‐controlled motor on the driving strings. This dual functionality of the driving string is essential for both the deployment process and securing the structure during transit (Figure 6D). Upon achieving the intended orbit, the actuation mechanism, by releasing the driving string, initiates the unfolding of the structure. This unfolding can be precisely controlled to pause at any specific intermediate state, enhancing its utility in varying space conditions.

## 3 Conclusion

The introduction of a deployable‐retractable annular structure marks a significant leap forward in the quest to mitigate space debris in low Earth orbit. Designed with medium‐sized debris in mind, this innovative system combines active contraction and mesh fabric to effectively capture and clear debris, providing a safer environment for satellites and other spacecraft. Its utility, however, extends far beyond debris removal. By adjusting the repeating units or substituting the mesh fabric with other materials, the structure can be repurposed for a variety of applications, such as expansive space‐deployable antennas or solar panel arrays, showcasing its remarkable adaptability.^[^ ^28^ ^]^

While the structure demonstrates promising capabilities, experimental assessments have highlighted specific areas in need of refinement, including stress concentrations at node junctions and the constraints related to the gear section's pitch circle diameter. The deployment strategy of this structure can be further improved and optimized for different environments. For instance, using pneumatic or electromagnetic drives could enable a more controlled unfolding process.^[^ ^29^ ^]^ Shape memory materials may allow the structure to expand or contract automatically in response to temperature changes.^[^ ^30^ ^]^ Additionally, by studying natural deployment mechanisms in plants and animals, we can develop flexible, self‐deploying strategies.^[^ ^31^ ^]^ Combining lightweight, high‐strength materials with pneumatic or electromagnetic drives can further enhance stability during self‐deployment.^[^ ^32^ ^]^ Furthermore, optimization algorithms can aid in planning optimal paths for deployment.^[^ ^33^ ^]^ Taken together, these approaches offer substantial opportunities for further optimization.

One of the structure's most compelling attributes is its scalability, which, when combined with a high deployment ratio, lightweight architecture, and robust load‐bearing capacity, renders it an ideal candidate for myriad deployable systems. The foundational principles of this design also lend themselves to miniaturization, paving the way for its application in fields as varied as civil infrastructure, medical devices, and beyond. This deployable technology represents a significant breakthrough, embodying a harmonious blend of functionality, adaptability, and ingenuity. It stands at the precipice of making substantial impacts across a spectrum of sectors, not limited to aerospace. Through its innovative design and potential for widespread application, this structure is poised to advance deployable technologies into new realms of possibility and practicality.

## 4 Experimental Section

### Materials

The prototype of the tensegrity structure was designed according to the ratio of 1:20, and its appearance is shown in Figure 5A. The height of the structure in the state of complete folding was 350 mm, and the diameter was ≈130 mm; in the state of complete opening, the height was ≈200 mm, and the diameter was ≈770 mm. The folding ratio was close to 1:6. It can be self‐driven, and the driving rope can be controlled to fix the structure in any intermediate state. The specific assembly requirements and experimental methods are as follows:

### Five‐Bar Linkage Node

First, two Limited blocks were assembled to the corresponding position of the Cover plate (outside), and then two Bar connections were respectively assembled to the corresponding shaft under the Limited block. The torsion spring was fixed to the shaft at the lower end of the Bar connection, with one end supported in the groove at the lower end of the Bar connection, and one end supported in the groove at the lower end of the Cover plate (outside). Finally, the Cover plate (inside) was assembled and fixed with bolts and nuts of corresponding size. The metal pulley was fixed at the top position of the Cover plate (inside), and 5.5 mm stainless steel copper groove wheel was used as the pulley. The assembly effect is shown in Movie S1 (Supporting Information).

### Three‐Bar Linkage Node

The bevel gear connecting bar1 and bar2 was symmetrically assembled to the Synchronizing node cover (inside), and the two connecting bars were checked for synchronous rotation. Finally, the Synchronizing node cover (outside) was assembled and fixed with bolts. The assembly effect is shown in Movie S2 (Supporting Information).

### Vertical Bar

A vertical bar was taken, and its unopened part was fixed at the junction of the Three‐bar linkage node, and fixed with bolts and nuts. Select a spring with a wire diameter of 0.5 mm, diameter of 10 mm, length of 130 mm, and common spring steel material, and enter it from the opening of the vertical bar. Put in the slider to compress and drive the spring to expand and contract. Finally, connect the opening with the five‐bar linkage node, and fix it with bolts and nuts. The assembly effect is shown in Movie S3 (Supporting Information).

### Structure's Assembly

Use two integral vertical bars in staggered order and connect the bar connection and bevel gear connecting bar to the crossbar. In this way, a double‐layer folding structure with positive 12 deformation will be formed. Use ordinary kite lines to connect the slider with the diagonal five‐bar linkage node, so that the spring can play a role and self‐drive the structure to expand. Finally, connect the kite line at the pulley of the five‐bar linkage node of the structure as the driving rope. Install the net cloth at one part of the folding structure. At this point, the whole structure will be successfully assembled.

### Methods—Drive Test

The driving rope will be extended and connected with the hand winch. The length of the driving rope was contracted by manual control, with a rate of ≈1 mm s^−1^. In a quasi‐static environment, the force change and the length change of the compression spring in the driving rope were measured. After measuring multiple sets of data, the average value was obtained to get the result. After data processing, the length change of the compression spring was converted into the force change. The folding effect is shown in Movie S4 (Supporting Information).

### Methods—Load Test

When the control structure was in the state of expansion, 50, 100, 200, 300, and 500 g weights were respectively thrown vertically onto the mesh from the height of 1 m away from the net cloth (Movie S5, Supporting Information). When the control structure was in the state of expansion, a 100 g weight fell from 0.5, 1.0, 1.5, 2.0, and 2.5 m in the air respectively, and vertically impacted the mesh (Movie S6, Supporting Information).

To facilitate assembly and experimentation, most of the structural parts were made of 3D‐printed resin materials. Since the stiffness and accuracy of the resin materials were not as good as metal materials such as aluminum alloy, this material specifications unavoidably have some adverse effect on the experimental results. However, in practical applications, it was possible to consider replacing the core parts with stronger metal materials to significantly improve load‐carrying capacity and impact resistance.

## Conflict of Interest

The authors declare no conflict of interest.

## Author Contributions

L.Y.Z. and C.C.C. designed and supervised the research. E.D.S., A.L., and L.Y.Z. performed research. E.D.S., M.S.I, L.Y.Z., and C.C.C. analyzed data. E.D.S., A.L., M.S.I, L.Y.Z., and C.C.C. wrote the paper.

## Supporting information



Supporting Information

Supplemental Movie 1

Supplemental Movie 2

Supplemental Movie 3

Supplemental Movie 4

Supplemental Movie 5

Supplemental Movie 6

## Data Availability

Data sharing is not applicable to this article as no new data were created or analyzed in this study.
