# Supplementary material for: Adaptive Deployable Structure Enabled by Actively Controlled Tensegrity for Space Debris Removal
Source: Adv Sci (Weinh). 2025 Feb 14;12(14):2408617. doi: 10.1002/advs.202408617 (PMC11984902; doi:10.1002/advs.202408617)
Supplement: Supplementary file 1 — Supporting Information [file ADVS-12-2408617-s006.pdf]

ADVANCED SCIENCE
Open Access

Supporting Information

for *Adv. Sci.*, DOI 10.1002/advs.202408617

Adaptive Deployable Structure Enabled by Actively Controlled Tensegrity for Space Debris Removal

Endong Shang, Ao Li, Md Shariful Islam, Li-Yuan Zhang and Changyong (Chase) Cao**

Supporting Information

Adaptive Deployable Structure Enabled by Actively Controlled Tensegrity for Space Debris Removal

Endong Shang, Ao Li, Md Shariful Islam, Li-Yuan Zhang, and C. Chase Cao**

This PDF file includes:

Model for Deployment Kinematics
Tables S1 to S3
Legends for Movies S1 to S6

Other supporting materials for this manuscript include the following:

Movies S1 to S6

Model for Deployment Kinematics

The unfolding process can be mathematically described by establishing the positional relationships of various points within the deployable unit, relative to the chosen coordinate system. These relationships hinge on the lengths of *r* and *h*, which serve as the primary variables governing the spatial arrangement and movement dynamics of the structure. Through differential equations or geometric analysis, we can derive expressions that encapsulate the evolution of point positions as a function of *r* and *h*. This analytical framework enables the prediction of the structure's behavior under different unfolding scenarios, facilitating optimization of the deployment mechanism for efficiency and reliability. By adjusting *r* and *h* within their operational ranges, we can fine-tune the deployment dynamics to achieve desired spatial configurations, accommodating various mission requirements and environmental conditions.

$$A_i = \left(\frac{r}{2v \cdot \sin\frac{\pi}{v}} \cdot \cos\frac{2\pi}{v}(i-1), \frac{r}{2v \cdot \sin\frac{\pi}{v}} \cdot \sin\frac{2\pi}{v}(i-1), 0 \right) \tag{S1}$$

$$B_i = \left(\frac{r}{2v \cdot \sin\frac{\pi}{v}} \cdot \cos\left[\frac{2\pi}{v}(i-1)+\frac{\pi}{v}\right], \frac{r}{2v \cdot \sin\frac{\pi}{v}} \cdot \sin\left[\frac{2\pi}{v}(i-1)+\frac{\pi}{v}\right], a \cdot \sin\alpha \right) \tag{S2}$$

$$C_i = \left(\frac{r}{2v \cdot \sin\frac{\pi}{v}} \cdot \cos\left[\frac{2\pi}{v}(i-1)+\frac{\pi}{v}\right], \frac{r}{2v \cdot \sin\frac{\pi}{v}} \cdot \sin\left[\frac{2\pi}{v}(i-1)+\frac{\pi}{v}\right], a \cdot \sin\alpha + h \right) \tag{S3}$$

$$D_i = \left(\frac{r}{2v \cdot \sin\frac{\pi}{v}} \cdot \cos\left[\frac{2\pi}{v}(i-1)+\frac{\pi}{v}\right], \frac{r}{2v \cdot \sin\frac{\pi}{v}} \cdot \sin\left[\frac{2\pi}{v}(i-1)+\frac{\pi}{v}\right], a \cdot \sin\alpha + b \right) \tag{S4}$$

$$E_i = \left(\frac{r}{2v \cdot \sin\frac{\pi}{v}} \cdot \cos\frac{2\pi}{v}(i-1), \frac{r}{2v \cdot \sin\frac{\pi}{v}} \cdot \sin\frac{2\pi}{v}(i-1), b \right) \tag{S5}$$

Tables S1 to S2

Table S1. Space debris distribution and relative risks in different orbits.

Debris size		1-3 mm	4-9 mm	1-9 cm	≥10 cm	≥1 m
Debris quantities		100,000,000	10,000,000	500,000	19,000	2,000
Tracking status		Nontrackable	Nontrackable	Not actively tracked	Actively traeked and cataloged	
**RELATIVE RISK**	LEO	Very high	High	Moderate	Very low	Very low
	MEO	High	Moderate	Moderate	Very low	Very low
	GEO/GSO	High	Moderate	Low	Very low	Very low

Table S2. Impact tests under different impacting masses on the tensegrity ring.

Weight mass (g)	Sloshing	Passive folding	Mechanism damage	Overall evaluation
50	No	No	No	Very good
100	Slight	No	No	Very good
200	Slight	Slight	No	Good
300	Obvious	Slight	No	Good
500	Obvious	Obvious	Mesh and node disconnected	Common

Table S3. Impacts tests under different impacting speeds on the tensegrity ring.

Height mass (m)	Effective speed (m s^{-1})	Sloshing	Passive folding	Mechanism damage	Overall evaluation
0.5	3.2	Slight	No	No	Very good
1.0	4.5	Slight	No	No	Very good
1.5	5.5	Obvious	Slight	No	Very good
2.0	6.3	Obvious	Slight	No	Good
2.5	7.1	Obvious	Obvious	No	Good

Legends for Movies S1 to S5

Movie S1. Five-bar Linkage Node

The Five-bar linkage node consists of the following components: Cover plate (inside), Cover plate (outside), limited block, bar connection, torsion spring, pulley, and fastener. The bar connection links cross bars. The limited block restricts the development angle of the crossbar to prevent excessive internal forces in the structure. The torsion spring provides expansion force when the structure is fully folded (dead center). The drive rope passes through the pulley, significantly reducing sliding friction and making the folding process smoother. The reserved hole at the lower end of the Five-bar linkage node connects to the vertical bar.

Movie S2. Three-bar Linkage Node

The Three-bar linkage node consists of the following components: Synchronizing node cover (inside), Synchronizing node cover (outside), bevel gear connecting bar 1 & 2, and fastener. Bevel gear connecting bars 1 & 2 mesh with each other, connecting two crossbars respectively, allowing them to maintain a symmetrical angle in the same direction and size. The reserved hole at the lower end of the Three-bar linkage node connects to the vertical bar.

Movie S3. Unit Fold Display

The Unit fold display consists of the following components: Five-bar linkage node, Three-bar linkage node, spring, sliding block, and fastener. The spring is assembled inside the vertical rod to limit its compression trajectory and avoid instability. The sliding block connects to the diagonal rope and transmits the pressure of the spring to the entire structure, forcing the structure to unfold. The whole ring structure is composed of 12 Unit fold displays and 24 crossbars.

Movie S4. Repeatable Folding Performance

After assembling the overall structure, we extend the drive rope and connect it to a manual winch. By turning the winch, the drive rope contracts at a controlled speed of 1 cm/s, achieving a quasi-static contraction and expansion process. The repeated folding performance of the structure was tested under two conditions: slow contraction and slow release, and slow contraction and fast release. It was found that the latter condition achieves a greater degree of folding. The reason for this is that in actual operation, the structure's connections are not perfectly smooth, resulting in internal friction. The latter condition, being non-quasi-static, incorporates inertia force, allowing for a greater degree of repeated folding.

Movie S5. Impact Resistance of Different Masses

In the actual working environment, the mesh needs to withstand the impact of space debris. Therefore, impact resistance tests were conducted. When the control structure is in an expanded state, weights of 50 g, 100 g, 200 g, 300 g, and 500 g were dropped vertically onto the net cloth from a height of 1 m. It was found that the structure was near collapse when subjected to the impact of 500 g weights. According to the 1:20 structure ratio, it can withstand the impact of space debris with a mass of 50 g and a speed of 800 $m\ s^{-1}$. The test structure is made of resin, and using lightweight, high-strength aerospace materials would increase the actual load capacity by more than 10 times.

Movie S6. Impact Resistance at Different Speeds

In the actual working environment, the mesh may encounter debris impacts at varying speeds. To simulate these conditions, 100 g weights were dropped from heights of 0.5 m, 1 m, 1.5 m, 2 m, and 2.5 m onto the mesh to represent impacts at different speeds. The results demonstrate that the structure can passively fold and dissipate the energy of high-speed impacts, effectively reducing potential damage.
